# Differential Contribution of Anterior and Posterior Midcingulate Subregions to Distal and Proximal Threat Reactivity in Marmosets

**DOI:** 10.1093/cercor/bhab121

**Published:** 2021-06-01

**Authors:** Sufia S Rahman, Kevin Mulvihill, Christian M Wood, Shaun K L Quah, Nicole K Horst, Hannah F Clarke, Gemma J Cockcroft, Andrea M Santangelo, Angela C Roberts

**Affiliations:** Department of Physiology, Development and Neuroscience, University of Cambridge, Cambridge CB2 3DY, UK; Behavioural and Clinical Neuroscience Institute, University of Cambridge, Cambridge CB2 3EB, UK; Department of Physiology, Development and Neuroscience, University of Cambridge, Cambridge CB2 3DY, UK; Department of Physiology, Development and Neuroscience, University of Cambridge, Cambridge CB2 3DY, UK; Behavioural and Clinical Neuroscience Institute, University of Cambridge, Cambridge CB2 3EB, UK; Department of Physiology, Development and Neuroscience, University of Cambridge, Cambridge CB2 3DY, UK; Behavioural and Clinical Neuroscience Institute, University of Cambridge, Cambridge CB2 3EB, UK; Department of Psychology, University of Cambridge, Cambridge, UK; Behavioural and Clinical Neuroscience Institute, University of Cambridge, Cambridge CB2 3EB, UK; Department of Physiology, Development and Neuroscience, University of Cambridge, Cambridge CB2 3DY, UK; Behavioural and Clinical Neuroscience Institute, University of Cambridge, Cambridge CB2 3EB, UK; Department of Physiology, Development and Neuroscience, University of Cambridge, Cambridge CB2 3DY, UK; Behavioural and Clinical Neuroscience Institute, University of Cambridge, Cambridge CB2 3EB, UK; Department of Physiology, Development and Neuroscience, University of Cambridge, Cambridge CB2 3DY, UK; Behavioural and Clinical Neuroscience Institute, University of Cambridge, Cambridge CB2 3EB, UK; Department of Physiology, Development and Neuroscience, University of Cambridge, Cambridge CB2 3DY, UK; Behavioural and Clinical Neuroscience Institute, University of Cambridge, Cambridge CB2 3EB, UK

**Keywords:** anxiety, cingulate cortex, emotion regulation, human intruder test, Pavlovian threat conditioning

## Abstract

The midcingulate cortex (MCC) is associated with cognition and emotion regulation. Structural and correlational functional evidence suggests that rather than being homogenous, the MCC may have dissociable functions that can be mapped onto distinct subregions. In this study, we use the marmoset monkey to causally investigate the contributions of two proposed subregions of the MCC: the anterior and posterior midcingulate cortices (aMCC and pMCC) to behavioral and cardiovascular correlates of threat processing relevant to anxiety disorders. Transient inactivation of the aMCC decreased anxiety-like responses to a postencounter distal threat, namely an unfamiliar human intruder, while inactivation of the pMCC showed a mild but opposing effect. Furthermore, although inactivation of neither MCC subregions had any effect on basal cardiovascular activity, aMCC inactivation blunted the expression of both cardiovascular and behavioral conditioned responses to a predictable proximal threat (a rubber snake) during the extinction in a Pavlovian conditioning task, with pMCC inactivation having again an opposing effect, but primarily on the behavioral response. These findings suggest that the MCC is indeed functionally heterogeneous with regards to its role in threat processing, with aMCC providing a marked facilitative contribution to the expression of the emotional response to both proximal and distal threat.

## Introduction

The dorsal anterior cingulate cortex (dACC) has been implicated in negative emotion and emotion regulation (Mechias et al. 2010; Etkin et al. 2011; Shackman et al. 2011). By virtue of its structural connectivity with both higher-order cortical association regions of the brain and limbic regions, it has been hypothesized to act as a key node for the integration of cognition and emotion (Drevets et al. 2008; Stevens et al. 2011; Ray and Zald 2012). Indeed, functional neuroimaging studies in healthy individuals have implicated the dACC in a vast array of both cognitive functions, such as decision-making, error processing and conflict monitoring (Botvinick 2007; Ullsperger et al. 2014; Kolling et al. 2016), and affective functions, such as fearful and anxious behavior (Milad et al. 2007; Shackman et al. 2011) as well as sympathetic arousal (Critchley et al. 2003). Impairments in both cognitive and emotional domains are apparent in affective disorders, and abnormalities in the structure and function of the dACC have been observed in anxiety (Etkin and Wager 2007; Shin et al. 2009) and depression (Caetano et al. 2006; Koolschijn et al. 2009; Hamilton et al. 2012), suggesting a role for the dACC in the pathophysiology of these disorders.

The dACC, however, is a large heterogeneous region composed of a number of cytoarchitectonically defined subdivisions that lie in front of and behind the genu of the corpus callosum. Human neuroimaging studies would suggest that the dACC is also functionally heterogeneous (Drevets et al. 2008; Vogt 2016). Indeed, based on the parcellation of Vogt, the dACC can be separated into a region anterior to the genu of the corpus callosum, pregenual (p)ACC and a more caudal region, the midcingulate cortex (MCC) (Vogt et al. 2003). This parcellation also distinguishes an anterior and posterior subdivision (aMCC, pMCC) within the MCC. All three regions can be differentiated according to not only a variety of cyto- and chemoarchitectural characteristics and connectivity patterns (Vogt et al. 2003) but also the differential activity patterns across these three regions described by neuroimaging studies as reviewed in Vogt (2016). One particular prominent feature of their connections that differentiates them is the greater connectivity between the aMCC and the amygdala and the pMCC with the posterior parietal cortex, something we will return to in the discussion. Of particular relevance to our understanding of threat processing is the finding that negative emotion–related activity is primarily observed in the aMCC and not the pMCC or pACC (Büchel et al. 1998; Milad et al. 2007; Mechias et al. 2010; Vogt 2016). Moreover, this same region is implicated in sympathetic arousal including cardiovascular activity (Buchanan and Powell 1982; Critchley et al. 2003), which is a core component of negative affect in response to threat. However, functional imaging findings are correlational and may not infer causality. Thus, in order to determine the possible rostrocaudal functional differentiation within the MCC with respect to negative emotion, targeted manipulations in experimental animals are required.

There have been a number of intervention studies of dACC in rodents investigating its role in anxiety and conditioned fear (Bissiere et al. 2006; Vidal-Gonzalez et al. 2006; Bissière et al. 2008). In many cases, however, alternative nomenclature is used in these studies (ACd: dorsal anterior cingulate; rCG1/rCG2: rostral anterior cingulate cortex) and it is not always clear how the regions targeted map onto the MCC as defined by Vogt. It is common in rodents to refer to cg1 and cg2 as dACC (Preuss 1995; Ongur and Price 2000; Heilbronner and Hayden 2016) and these regions roughly correspond to the MCC in the parcellation of the rodent dACC by Vogt (Vogt and Paxinos 2014). It should be noted though that while the MCC in humans has anterior and posterior subdivisions, the putative comparable region in rodents is relatively uniform (Van Heukelum et al. 2020), with both anterior and posterior extents showing similar connectivity patterns (Fillinger et al. 2017, 2018). Moreover, comparative functional studies of Pavlovian conditioned threat and extinction in humans and rodents have led to the alternative proposal that it is the rodent prelimbic region that is analogous to the human dACC, and in particular the MCC (Milad and Quirk 2012).

In contrast to the rodent, the parcellation of the nonhuman primate (NHP) cingulate cortex appears more comparable to that of humans in the extent to which there is an anterior and a posterior subdivision of the MCC (Vogt et al. 2005). However, fewer intervention studies in NHPs have taken place, and in many cases, they targeted the whole extent of the dACC, including parts of the MCC, with social interactions and decision-making being the behavioral focus (Hadland et al. 2003; Chudasama et al. 2013). More selective targeting has compared the cingulate sulcus and gyrus, but their lesion areas are primarily located anterior to the genu of the corpus callosum (Kennerley et al. 2006; Rudebeck et al. 2006) including in the latter, area 32. Moreover, the above studies used the ablation technique, making it difficult to rule out that some of the effects were not due to disruption of fibers of passage. In the one study investigating specifically the MCC, using low-frequency stimulation to induce temporary inactivation, this region was implicated in conditioned threat regulation (Klavir et al. 2012) but the target region appeared to span both the anterior and posterior MCC subdivisions.

In order to dissect out the potential differences in function across the anterior–posterior subdivisions of the MCC with respect to the regulation of negative emotion, the present study compared these two subregions in the New World monkey, the common marmoset. While the dACC of the marmoset has not been formally parcellated into pACC, aMCC, and pMCC, these subdivisions can be distinguished according to the cyto- and chemoarchitecture described in humans and macaques (Vogt et al. 2003, 2005) using, for example, neurofilament protein expression (Paxinos et al. 2012), which is confirmed in the current study. Here, we evaluate threat processing under the framework of the predatory imminence hypothesis (Perusini and Fanselow 2015), using a variety of different contexts in which threat is either proximal or distal, highly translatable to studies of anxiety in humans. According to this framework (Perusini and Fanselow 2015), negative emotions can be seen as responses along a continuum of threat across time (the here and now, vs. sometime in the future), space (near vs. far) and probability (certain vs uncertain). Depending upon the proximity of the threat and how much time is available, different behaviors, cognitions, and emotions are engaged (Mobbs et al. 2020). It is proposed that anxiety is associated with more distal threat, while fear is associated with more proximal threat. Indeed, the MCC, including aMCC, has been specifically implicated in more proximal threat in recent neuroimaging studies that have varied threat levels along this continuum (Qi et al. 2018). Thus, here we investigated the causal contribution of the putative marmoset MCC subregions, aMCC and pMCC, to the regulation of threat processing by assessing the effects of transient, selective inactivation on the behavioral responses to postencounter distal threat (human intruder – HI test) and cardiovascular and behavioral responses to predictable circa-strike threat (Pavlovian threat conditioning and extinction paradigm). The latter has been used effectively to assess the ability to flexibly regulate threat responses when there are changes in the relationship between threat and the stimuli that predict it (reviewed in Milad and Quirk 2012). Persistent expression of threat responses to stimuli that no longer predict threat is reported in, for example, posttraumatic stress disorder (reviewed in VanElzakker et al. 2014) and neuroimaging studies reveal altered processing in anxiety disorders (Marin et al. 2017).

Given that a major characteristic of mood and anxiety disorders is alterations in cardiovascular activity (reviewed in Celano et al. 2016), the contribution of MCC subregions to basal cardiovascular activity was also measured in an affectively neutral condition, acting in addition as a control for any manipulation effects on threat-induced cardiovascular responses. Based on the neuroimaging studies described above, we hypothesized that inactivation of the anterior, but not the posterior, MCC would induce a reduction in threat reactivity to proximal threat, but whether it would also impact on reactivity to distal threat has yet to be determined.

## Materials and Methods

### Subjects

**Figure 1:**
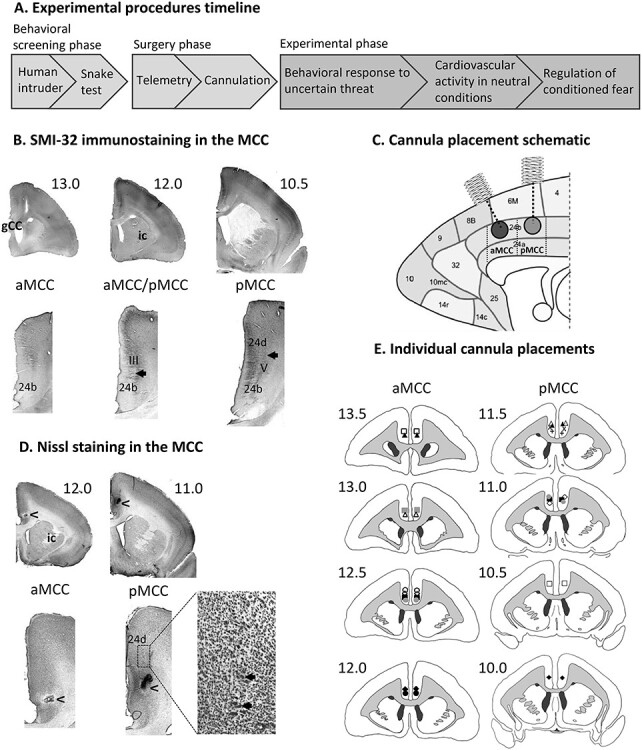
Experimental time line and cannula placements. (*A*) Time line of experimental procedures. (*B*) Coronal sections showing the histological parcellation of aMCC (left panel), and pMCC with SMI-32–positive neurons in layer III of area 24b (middle panel) and layer V of area 24d (right panel). (*C*) Schematic representation of the cannula placements, sagittal midline section adapted from Burman and Rosa 2009. MCC subregion boundaries marked with dashed lines are approximate. (D) Coronal Nissl-stained sections showing examples of cannula placements (arrowheads) in aMCC (left panel) and in pMCC (right panel), with large pyramidal neurons in layer V of area 24d (inset). (E) Individual cannula placement locations drawn on coronal section schematics. Symbols correspond to individual marmosets consistent with those used throughout the manuscript.

A total of 12 marmosets (*Callithrix jacchus*, six females and six males) were used in the present study (Table 1). Marmosets were screened in early adulthood using the HI and rubber snake tests to assess their behavioral responsivity to threat, as previously described (Shiba et al. 2014). With the exception of three (Tr, W-e, and S) who had received structural MRI scans as infants as part of an independent neurodevelopmental study, all marmosets had no previous experimental procedures. A time line of experimental procedures included in this study is provided in Figure 1*A*. There were no manipulations performed in the affectively neutral condition for Wa due to human error. Ye, W-e, Bu, and Ju did not participate in the threat Pavlovian conditioning task. Ye and W-e had telemetry probe failures and Bu and Ju were allocated to a different study after the HI. See Table 1 for subjects’ participation in each experiment. All marmosets were bred on site at the Innes Marmoset Colony (Behavioral and Clinical Neuroscience Institute) and housed in male/female pairs (males were vasectomized), under temperature- (22 ± 1 °C) and humidity- (50 ± 1%) controlled conditions in purpose-built cages. A variety of environmental enrichment aids were provided including beams, branches, and ropes. A 12-h dawn-/dusk-like light–dark cycle was maintained and marmosets were provided with a varied and balanced diet with water available ad libitum. All procedures were performed in accordance with the UK Animals (Scientific Procedures) Act 1986, the personal and project licenses, and the AWERB policies.

**Table 1:** Detail of subjects’ participation across experimental settings. All animals were included in the study measuring the effects of MCC subregions’ inactivation on behavioral responses to distal threat (HI test; *N* = 12). A subset was involved in assessment of MCC subregions’ inactivation on basal cardiovascular activity during affectively neutral conditions (*N* = 9), and an overlapping subset, in regulation of conditioned threat (*N* = 8). See Materials and Methods for further details

Subject	Behavioral response to distal threat	Cardiovascular activity in neutral conditions	Regulation of conditioned threat	Area	Symbols
Ja	√	√	√	aMCC/pMCC	∆
To	√	√	√	aMCC/pMCC	⊗
G	√	√	√	pMCC	+
Ba	√	√	√	aMCC/pMCC	□
Tr	√	√	√	aMCC	○
Wa	√		√	aMCC/pMCC	♦
S	√	√	√	aMCC/pMCC	◊
A	√	√	√	aMCC/pMCC	■
W-e	√	√		aMCC	●
Ye	√	√		pMCC	×
Ju	√			aMCC	$\boxtimes$
Bu	√			aMCC/pMCC	▲
Total	12	9	8		

Note: Symbols ⊗ and $\boxtimes$ shown in gray in Figures 1 and 4.

### Determination of Target Coordinates for Cannulation

Histological material from an additional marmoset (unpublished data) was used to assess neurofilament protein (NFP) SMI-32 staining within the MCC region of area 24 as an aid to demarcating the anterior and posterior subdivisions of the MCC. SMI-32 staining was performed using a protocol identical to the one used in Paxinos et al. (2012). Vogt et al. (2003) have shown in humans and macaques that layers III and V of the pMCC are relatively thicker, with a greater density of neurons, and more NFP-expressing neurons than the aMCC. Based on both the cytoarchitectural description of the MCC in macaques (Vogt et al. 2003) and the SMI-32 immunostaining shown in the Marmoset Atlas (Paxinos et al. 2012), we established two subregions within the marmoset MCC: aMCC spanning from the appearance of the genu to just after the appearance of the internal capsule (ic), with no SMI-32 staining observed either in layers III or V within area 24b (Fig. 1*B*, left panel); and pMCC spanning from the appearance of SMI-32–positive large neurons in layer III of area 24b, where the ic is well distinguishable (Fig. 1*B*, middle panel) to just before the appearance of the anterior commissure, where a dysgranular area 23d with clumps of neurons in layer IV and a very dense layer V can be observed. Within the center of the rostrocaudal extent of pMCC, SMI-32–positive motoneurons are also observed in layer V of area 24d (Fig. 1*B*, right panel). Thus, the Paxinos coordinates were established to target the middle extent of these two regions. See schematic of cannula placements in Figure 1*C*.

In addition, the above-mentioned SMI-32–positive large neurons in layers III (area 24b) and V (area 24d) can be easily observed using Nissl staining (Fig. 1*D*), so we determined the cannula placements by observing Nissl-stained sections (see below).

### Surgical Procedures

Subjects were premedicated with ketamine hydrochloride (Vetalar; 0.05 mL of a 100-mg solution, i.m.; Amersham Biosciences and Upjohn) and given a long-lasting nonsteroidal anti-inflammatory analgesic agent (Carprieve; 0.03 mL of 50 mg/mL carprofen, s.c.; Pfizer). They were then intubated with an intratracheal tube attached to an anesthetic machine with anesthesia maintained at ~ 2.5% isoflurane in 0.3 L/min O_2_. Subjects lay on a heat mat. Pulse-rate, O_2_ saturation, respiratory rate, and CO_2_ saturation were monitored by pulse-oximetry and capnography (Microcap Handheld Capnograph, Oridion Capnography Inc., MA, USA), and core body temperature was monitored by a rectal thermometer (TES-1319 K-type digital thermometer, TES Electrical Electronic Corp., Taipei, Taiwan).

#### Telemetry

Subjects were anesthetized as described above. The telemetric blood pressure transmitter (PhysioTel implant, model PA-C40 or HD-S10; Data Sciences International [DSI], St. Paul, MN, USA) was placed in the abdomen and the catheter inserted into the descending aorta, as described previously (Braesicke et al. 2005). Subjects received meloxicam postsurgery and were also given prophylactic treatment of amoxicillin and clavulanic acid (Synulox; 50 mg/mL solution; Pfizer), for 1 day before and 6 days after telemetry surgery.

#### Cannulation

Subjects were anesthetized as described above and were positioned in a stereotaxic frame, especially modified for the marmoset (David Kopf, Surgical Instruments). Bilateral guide cannulae (26-gauge, 3.5 mm length, 2 mm center to center distance; Plastics One, Roanake, VA, USA) were implanted into the aMCC (AP +13.5 at 40° angle; LM ± 1, depth −3.3) and pMCC (AP +11.0, LM ± 1, depth −3.8) (Fig. 1*C*). Surgical coordinates were adjusted in situ if necessary to account for individual differences in forebrain size (Roberts et al. 2007). Once recovered from anesthesia, subjects were returned to their home cage and were given an analgesic (Meloxicam, 0.1 mL of a 1.5 mg/mL oral suspension; Boehringer Ingelheim, Germany) for 3 days postsurgery and were allowed at least 10 days for further recovery. Cannulation sites were cleaned, and dummy injectors and caps were changed for sterile ones at least once weekly to avoid occurrence of infection.

### Intracerebral Infusions

All experimental animals were habituated to the procedure prior to the start of experiments. The subject was caught by a researcher and held gently. Caps and dummy injectors were removed from the cannula guide and the site was cleaned with 70% alcohol. A sterile injector (length adjusted based on surgical coordinates to target area 24b) was attached to two gastight Hamilton syringes (10 μL, Sigma-Aldrich, Missouri, USA), which were placed in an infusion pump, and was inserted into the guide cannula. 0.5 μL of 0.9% sterile saline or 0.5 μL of 0.1 mM muscimol/1.0 mM baclofen was infused at a rate of 0.25 μL/min (Clarke et al. 2005). The injector was left in situ for a further minute after the infusion to allow for diffusion and then removed, and fresh sterile dummy injectors were inserted in the guide cannula and caps screwed on. The subjects were returned to their home cage for 20–25 min before testing.

### Assessment of Cannula Placements

Marmosets were transcardially perfused, with 400 mL of 0.1 M PBS, followed by 400 mL of 10% neutral buffered formalin (Sigma-Aldrich, Missouri, USA) as a fixative over ∼20 min. The entire brain was then dissected and placed in fixative solution for 12–24 h. The brain was transferred to a 30% sucrose/0.1 M PBS solution for at least 48 h for cryoprotection. The brain was frozen using crushed dry ice and mounted on a freezing microtome. Coronal brain sections (60 μm) were taken and stored in well plates filled with 0.01 M PBS at 4 °C. Every third section was mounted onto microscope slides and stained with Cresyl Fast Violet (Sigma-Aldrich, Missouri, USA). Stained sections were viewed under a Leitz DMRD microscope (Leica Microsystems, Wetzlar, Germany). Cytoarchitectural characteristics were observed on Nissl-stained sections to determine cannula placements within either aMCC or pMCC (Fig. 1*D*), and they were then schematized onto drawings of standard marmoset brain coronal sections (Fig. 1*E*).

### Human Intruder Test and Pharmacological Manipulation

**Figure 2:**
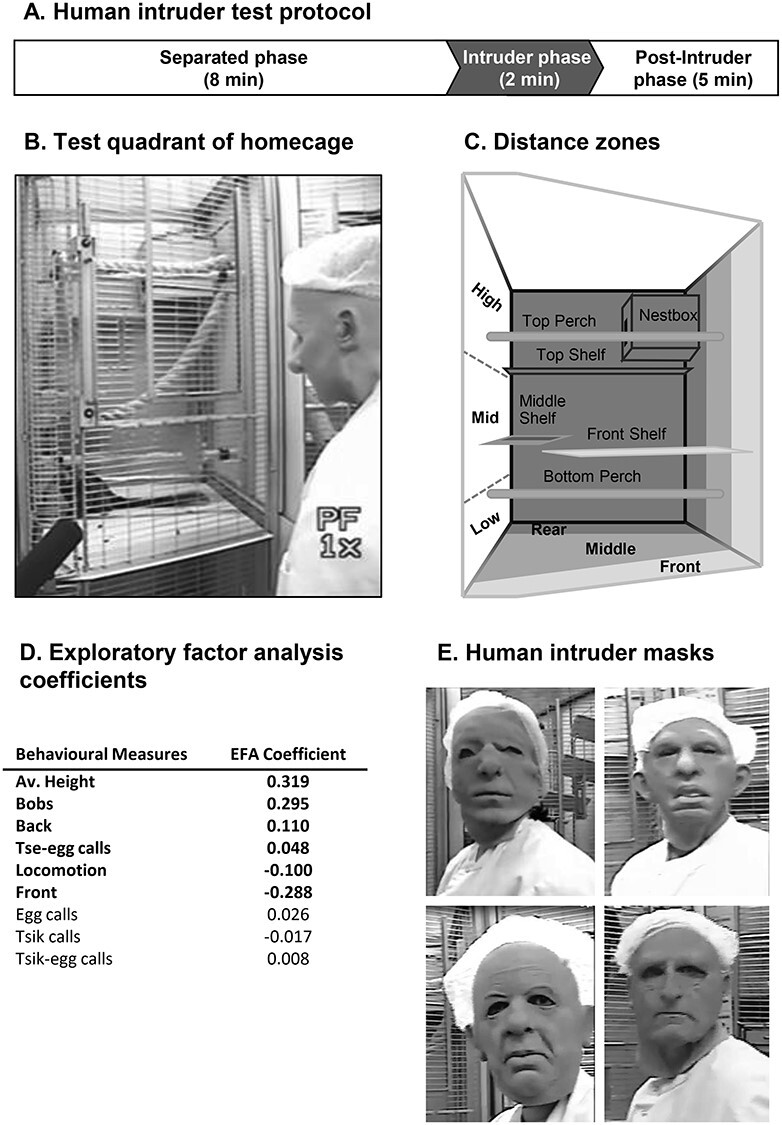
Experimental details of the HI test used to analyze postencounter distal threat. (*A*) Time line of HI test protocol. (*B*) Video still during intruder phase of the HI test. (*C*) Schematic of the top right quadrant of the home cage (i.e., test quadrant) showing division into zones for height and depth. (*D*) Behavioral measures that contribute to the exploratory factor analysis and their coefficients used to calculate the overall score of anxiety-like behavior. The measures contributing significantly are shown in black. (*E*) Examples of masks used by the HI.

The test was conducted as previously described (Santangelo et al. 2016). Briefly, the subjects were separated from their partner and divided into the right-hand upper quadrant of the home cage (separated phase, Fig. 2*A*). The subject’s behavior was recorded using a GoPro camera and a Sennheiser MKE 400 microphone, which were placed at a short distance from the cage (Fig. 2*B*). After 8 min, an unfamiliar experimenter, the “HI,” entered the room and stood 40 cm away in front of the cage. The HI was disguised using a realistic looking human mask (Masks Direct) and wearing familiar scrubs and gown. When possible, the intruder made direct eye contact with the subject for 2 min (intruder phase). The intruder then quietly left the room, and the subject’s behavior was recorded for a further 5 min (postintruder phase).

#### Behavioral Analysis

Behavioral scoring was done blind to cannula placements. A quantitative behavioral analysis program (Jwatcher V1.0) was used to score a range of different behaviors during the 2-min intruder phase from playback of video recordings of the HI test sessions. One of the main measures is the percentage time spent in various cage locations, taking into account both depth from the cage front and height (Fig. 2*C*). When anxious, marmosets tend to position themselves toward the back of the cage and to move higher. Upward flight has previously been reported as a defensive response, particularly to terrestrial predators (Barros 2002). Additional measures included locomotion, head and body bobbing (a marmoset-specific vigilance behavior displayed during uncertainty), and a variety of vocalizations (Stevenson and Poole 1976). A composite score that underlies anxious behavior has been derived from an exploratory factor analysis (EFA) performed on this range of behaviors displayed by a cohort of 171 naïve marmosets from the colony (Quah et al. 2020) (Fig. 2*D*). This revealed a single factor that accounts for 39.7% of the variance in the colony. The behavioral measures associated with this factor included proportion of time spent at the front and back of the cage, average height, locomotion, head and body bobs, and tsik, tsik-egg, tse-egg, and egg vocalizations. The pattern of behaviors clustering on this factor suggest that it represents the animal’s anxiety-like response toward the HI, with those animals with the highest score spending most of the time toward the back of the cage, high up, remaining relatively still, and making head and body bobs and calls.

#### Pharmacological Manipulations

To assess the effect of inactivation of MCC subregions on anxiety levels, an intracerebral infusion of saline or muscimol/baclofen was administered via implanted MCC-targeted cannula, 25 min before the test session, and we performed repeated HI tests (Santangelo et al. 2016), at least one week apart. The intruder wore different rubber masks during each session to give the appearance of a novel HI (Fig. 2*E*), which were counterbalanced between subjects. The HI test was repeated five times in the following order: saline, drug, saline, drug, saline. The counterbalanced order of the infusions into the MCC subregions is detailed in Supplementary Table 1.

#### Statistical Analysis

The factor correlation coefficients from the EFA (Quah et al. 2020) were used to calculate an overall anxiety score for each animal included in the current study standardized to the control vehicle conditions. Muscimol/baclofen inactivation of each MCC subregion was compared with the averaged values of the saline sessions occurring before and after the inactivation. For the EFA scores, mixed-model ANOVA was carried out using the statistical language and environment R (version 3.6.3; R Core Team, 2020) with the R Studio interface (version 1.2.5033; Rstudio, Inc.), using the lme4 package (Bates et al. 2015) for linear mixed-effects modeling, statistical tests from the lmerTest package, and type III sums of squares with the Satterthwaite approximation for degrees of freedom (here reported to the nearest integer). Subject was added as a random effect, Treatment (saline vs. muscimol/baclofen) and Area (aMCC vs. pMCC) as fixed effects. F values and *P* values are reported to three significant figures where appropriate. Pairwise comparisons were conducted using emmeans package (Lenth 2020) with Tukey adjustment to control familywise error rate with multiple comparisons. For the individual behavioral measures, a two-tailed paired *t*-test was used.

### Basal Cardiovascular Activity, Pavlovian Threat Conditioned, and Pharmacological Manipulations

#### Apparatus

**Figure 3:**
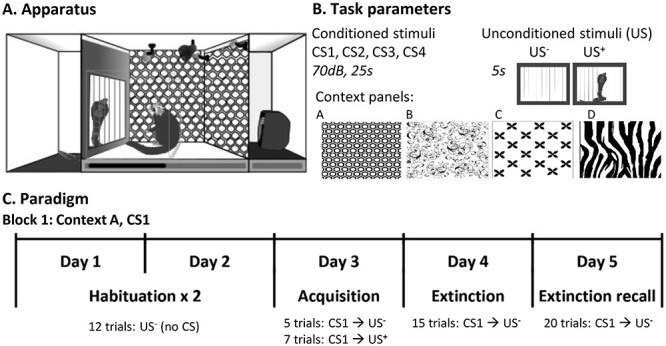
Experimental settings of the Pavlovian threat conditioning and extinction paradigm used to assess the regulation of conditioned threat. (*A*) Diagram of a marmoset in the testing apparatus during an acquisition trial in which the unconditioned stimulus (rubber snake; US^+^) is presented. (*B*) Schematic representation of the “task parameters” including the different auditory conditioned stimuli (CS; 70 db, 25 s) used across blocks (sounds: dream-harp, phone, bell, fish-tank; CS1–4), the different wall panels used to change the apparatus context (hexagon, swirly, crosses, zebra) in different blocks and the US- (black door opening for 5 s to reveal an empty chamber), the US^+^ (black door opening for 5 s to reveal a rubber snake). (*C*) Schematic showing an example block detailing the number of trials presented in each session.

Assessing basal cardiovascular activity and regulation of conditioned threat responses in the Pavlovian threat extinction task occurred in a custom-built automated apparatus (Fig. 3*A*), enclosed within a sound-attenuated box and located in a designated testing room. Subjects were trained to enter a transparent Perspex carry box that was used to transport the test subject from the home cage to the behavioral apparatus into which it was placed. The door to the carry box was removed to reveal cage bars and another chamber behind them. The test chamber was lit by LED strips (house light) and contained three video cameras (LICG24SM IR Color camera, Defender Security) through which the subject could be observed and recorded (Power Director, Cyberlink). An adjacent chamber contained a speaker through which auditory stimuli could be played (Logitech Z120 speakers). The apparatus was controlled by the Whisker control system (Cardinal and Aitken 2010).

#### Telemetry Data Collection and Analysis

The implanted telemetry probe continuously detected blood pressure from the marmoset and transmitted it to a receiver (RPC-1; DSI) located beneath the behavioral testing chamber for offline analysis using data acquisition and analysis software (Spike2 Version 8; CED) as described previously (Braesicke et al. 2005). Systolic and diastolic blood pressure (sBP; dBP) events were extracted from the blood pressure trace as local maxima or minima, respectively, and mean arterial pressure (MAP) calculated from these (sBP/3 + 2dBP/3). Interbeat intervals (IBIs) were measured as the time interval between successive systolic blood pressure events and heart rate was derived from this (HR = IBI/60). Data were processed to remove outliers (values outside the pressure range 20–200 mmHg, and the IBI range of 0.1–0.4).

### Basal Cardiovascular Activity in Affectively Neutral Conditions

To determine the contributions of the MCC subregions to basal cardiovascular activity, these regions were inactivated independently in a familiar, affectively neutral context. Subjects were first placed in the test apparatus with the house light “on” for 10- to 20-min daily sessions, to habituate them to the context. Habituation was defined as the point at which heart rate and blood pressure remained stable across three sessions, and the subject’s posture was relaxed, that is, no piloerection, no curled tail, normal amount of grooming (Stevenson and Poole 1976). This took an average of 11 sessions (min = 7, max = 17, SD = 3.2). Following habituation, subjects continued to receive daily test sessions, in which they were placed in the test apparatus for 20 min, with the house light on. On manipulation days, an intracerebral infusion of saline or muscimol/baclofen was administered via implanted cannula to the targeted aMCC or pMCC (counterbalanced) prior to the test session. These occurred a maximum of twice a week and were interspersed with a minimum of two infusion-free test sessions to ensure no “carryover” effects of the manipulations.

#### Data Analysis

Data from the first minute of each session were excluded as the offset period and the following 15 min were sampled (i.e., 60–960 s) for calculation of average blood pressure and HR. The IBI data were imported into Kubios HRV version 2.2 (Tarvainen et al. 2014) and were detrended using the smoothness priors method (lambda = 500) (Tarvainen et al. 2002). The RMSSD, the square root of the mean-squared standard deviation of the time difference between successive IBIs (a time-domain measure of HRV), was calculated and Poincaré (Lorenz) plots (a scatter plot of each IBI interval against the next one) were generated. The standard deviation of the points on the Poincaré plots, perpendicular to the line of identity (SD1), and the standard deviation of the points along the line of identity (SD2) were used to calculate the cardiac vagal index (CVI) and the cardiac sympathetic index (CSI) (Toichi et al. 1997).

### Pavlovian Threat Extinction Task

To investigate the roles of the different MCC subregions in the regulation of threat, a threat conditioning and extinction paradigm was used (Wallis et al. 2017), adapted from a classic rodent paradigm (Sierra-Mercado et al. 2011). The threat extinction paradigm was conducted in the same apparatus as used for assessment of the basal cardiovascular activity (Fig. 3*A*). Each subject received four blocks of five sessions, with each block representing a new round of Pavlovian conditioning and extinction. To minimize fear generalization across session blocks, the context was changed by using different patterned wall panels (Fig. 3*B*, *A*–*D*) and distinct auditory stimuli as the conditioned stimuli (CS^+^) (Fig. 3*B*, CS1–4) in each block. Wall panels and CS stimuli were counterbalanced across treatments. Each block consisted of 2 days of habituation to the new context, 1 day of acquisition of conditioned threat to the rubber snake, followed by 1 day of extinction and extinction recall (Fig. 3*C*).

In the first two sessions, subjects were habituated to the new context (patterns on the wall of the apparatus that changed with every experimental block) and the unconditioned stimulus (US^−^), the latter involving 12 US^−^ presentations of a black door opening for 5 s to reveal an empty chamber behind. In each of the two sessions, there were 12 US^−^ presentations with a variable intertrial interval (vITI) between each of 110–130 s. In the third session (acquisition), the CS, an auditory sound (25 s, 70 db) was introduced, the last 5 s of which coterminated with the US. Since the marmosets initially show an unconditioned response to novel sounds, the first five trials during the acquisition session allows the monkey to habituate to this sound, so we can measure the specific conditioned response that subsequently develops specifically due to the association of the CS with the threat. The CS is followed by a US^−^ (door opens to show an empty chamber) so that it can be subsequently directly compared with the CS^+^, which is identical in every way except that the rubber snake is presented to represent the US^+^. Snakes are predators of marmosets and are thus an evolutionarily conserved threatening stimulus in primates (Ohman and Mineka 2001), and rubber snakes have previously been shown to provoke threat responses in laboratory bred marmosets (Barros et al. 2002; Shiba et al. 2014). CSs were presented with a vITI of 110–130 s.

In the fourth session (extinction), 20 CS–US^−^ pairings were presented with a vITI of 60–80 s. An intracerebral infusion of saline or muscimol/baclofen was administered via implanted cannula to the aMCC or pMCC prior to this session, in order to assess the effect of inactivation on conditioned threat expression and extinction. In the fifth and final session (extinction recall), 15 CS–US^−^ pairings were presented with a vITI of 60–80 s to test for recall of threat extinction. Session blocks were carried out in pairs such that session blocks 1–2 and session blocks 3–4 involved infusions in the same brain area, with area counterbalanced across subjects. Blocks 1 and 3 were saline infusions (see Supplementary Table 2).

#### Data Analysis

Cardiovascular responses during presentation of the 20 s CS and the immediately preceding 20 s baseline (BL) were analyzed. MAP rather than HR was used as the main autonomic measure of conditioned threat as the former has been strongly linked to threat conditioning in prior work (Wallis et al. 2017).

For analysis of the acquisition session, CS (US^−^) trial 1 was excluded due to its novelty (novel CS presented), and CS (US^−^) trials 4–5 were averaged as these represent the CS responding prior to formation of the CS-US^+^ association (PreUS). CS (US^+^) trial 6 was excluded from the analysis due to the manual placement of the rubber snake in the chamber during this trial, which could potentially be heard as a novel sound and induce a short (2–4 s) cardiovascular and orienting response. The remaining trials were averaged in pairs (CS pairs) (Sierra-Mercado et al. 2011; Wallis et al. 2017) for all session blocks. In order to account for individual variation in absolute levels of MAP, CS pairs were normalized within-subject by subtracting the mean of trials 4 and 5 (PreUS, description above) from every one of the CS pairs for a given subject, for acquisition, extinction, and recall sessions (Wallis et al. 2017). This calculation produces a difference score representing the delta between the respective CS pair of interest and the basal levels prior to the formation of the CS–US^+^ association.

#### Behavioral Analysis in Threat Extinction Study

Behavioral scoring was done blind to cannula placements, offline from video recordings of the test sessions. Time spent expressing vigilant behaviors (VB) was scored both during the 20 s CS and the immediately preceding 20 s BL as for cardiovascular analysis. We calculated a CS-directed measure by subtracting BL from the CS period (CS–BL). These included vigilant scanning, attentive scanning of the surroundings accompanied by a tense body posture, often marked by a forward extension of the body or head (Mikheenko et al. 2010; Agustín-Pavón et al. 2012), and head-jerks, defined as rapid lateral movements of the head. The CS-directed VB data were normalized as described above for MAP, with PreUS subtracted from subsequent CS pairs for all sessions.

#### Statistical Analysis

Cardiovascular and vigilant behavior data were statistically analyzed by mixed-model ANOVA performed with R (version 3.6.3; R Core Team, 2020) with the R Studio interface (version 1.2.5033; Rstudio, Inc.) using the lme4 package for linear mixed-effects modeling (Bates et al. 2015) with type III sums of squares and the Satterthwaite approximation for degrees of freedom (reported to nearest integer). Normality was assessed via a Shapiro–Wilk test on the residuals of the model. In instances where normality could not be assumed, an aligned-rank transformation of the data was performed using ARTool package (Wobbrock et al. 2011). Following such a transformation, data were analyzed by an analysis of variance using type III sums of squares Wald *F* tests with Kenward–Roger approximation for degrees of freedom. Separate ANOVAs were performed for each phase across cardiovascular and behavioral measures with α controlled per ANOVA. Where applicable, area (aMCC vs. pMCC), treatment (saline vs. muscimol/baclofen), and trials (PreUS, CS pair 1 … CS pair *n*) were treated as fixed effects with subject added as a random effect. For extinction and recall phases, effects across the duration of the session (factor: Time) were first investigated as a two-level factor included in the omnibus analysis (first and second halves of respective phase). If an interaction was found with factors of interest, it was followed up using the full number of factor levels (10 and 8 trials—CS pairs—for extinction and recall, respectively). Pairwise comparisons were conducted using emmeans package (Lenth 2020) with Tukey adjustment to control familywise error rate with multiple comparisons.

## Results

### Inactivation of aMCC Reduced the Anxiety-Like Responses to Postencounter Distal (Uncertain), Threat in the Form of an Unknown Human

**Figure 4:**
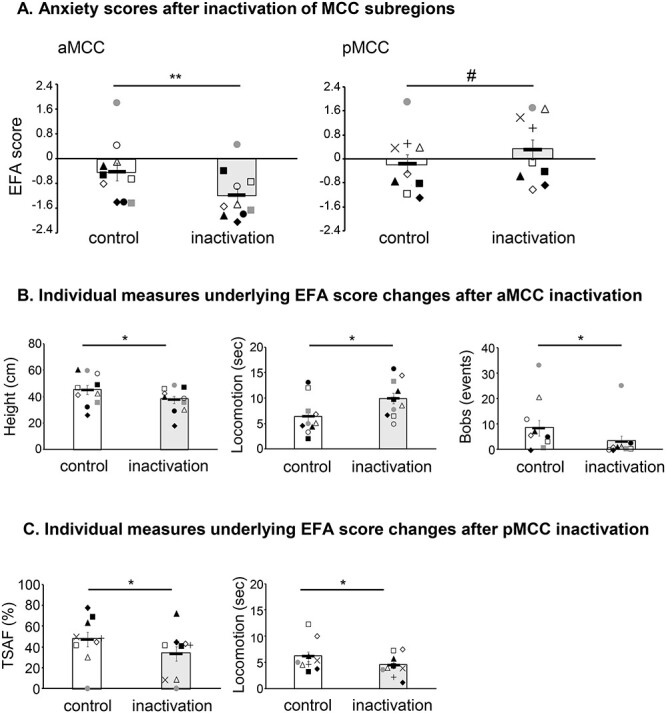
Effect of inactivation of MCC subregions on behavioral responses to postencounter distal threat in the form of an unknown human. (*A*) Inactivation of the aMCC (left) had an anxiolytic-like effect (reduction of EFA scores), while inactivation of the pMCC (right) showed a trend toward an anxiogenic-like effect (increase of EFA scores, #*P* = 0.058). (*B*) The anxiolytic-like effect after inactivation of aMCC was mainly driven by a decrease in height and bobs, and an increase in locomotion. (*C*) The anxiogenic trend observed after inactivation of the pMCC was evidenced by a reduction in time spent at the front (TSAF) near the HI and in locomotion, indicative of an avoidant response to threat. Symbols correspond to the ones used in Table 1. Linear mixed-model ANOVA, post hoc contrasts ^*^P < 0.05, ^**^P < 0.005, ^#^*P* = 0.058.

Inactivation of the aMCC during the HI test induced an anxiolytic-like effect evidenced by a significant reduction in the EFA anxiety score (Fig. 4*A*, left). The opposite, a mild anxiogenic-like effect was apparent following inactivation of the pMCC, with a trend for an increased EFA score (Fig. 4*A*, right). A linear mixed-model ANOVA revealed a significant treatment by area interaction (*F*_1, 22.97_ = 14.62; *P* = 0.001). Compared with saline, inactivation of the aMCC reduced anxiety-like behaviors (post hoc contrast *t*-ratio_23_ = −3.46; *P* = 0.002), while inactivation of the pMCC had a trend toward an increase in anxiety-like behaviors (post hoc contrast *t*-ratio_23_ = 1.99; *P* = 0.058).

To further determine the behavioral components that underpinned the differences in the composite anxiety scores between aMCC and pMCC after inactivation, additional analyses were performed on the individual behavioral measures of the HI test (see Supplementary Table 3). They revealed that while the anxiolytic-like effect of aMCC inactivation was mainly driven by a specific decrease in height and bobs, and an increase in locomotion (two-tailed, paired *t*-test control vs. inactivation, height: *P* = 0.014; bobs: *P* = 0.024; locomotion: *P* = 0.019; Fig. 4*B*), the mild anxiogenic-like effect of pMCC inactivation was driven by a reduction in both time spent at the front (TSAF) and locomotion, indicative of an avoidant response (two-tailed, paired *t*-test control vs. inactivation, TSAF: *P* = 0.046; locomotion: *P* = 0.017; Fig. 4*C*). During the preceding separated phase, only inactivation of the aMCC showed increased locomotion, with no effect on all other measures (see Supplementary Table 4).

### Inactivation of aMCC or pMCC Had No Effects on Measures of Basal Cardiovascular Function

Inactivation of either aMCC or pMCC had no effects on cardiovascular function in an affectively neutral environment. A linear mixed-model ANOVA showed no significant main effects or interactions for any of the measures of cardiovascular function, namely HR, MAP, or HRV measures (RMSSD, CSI, CVI, CVI/CSI). All *F* < 1, except for MAP (*F*_1,15.89_ = 3.61, *P* = 0.076) and CSI (*F*_1,13.89_ = 4.06, *P* = 0.064) for main effect of treatment, and HR (*F*_1,16.09_ = 1.08, *P* = 0.310), RMSSD (*F*_1,13.89_ = 1.07, *P* = 0.320), and CVI/CSI (*F*_1,13.82_ = 2.20, *P* = 0.160) for area by treatment interaction. See Table 2 for the results.

**Table 2:** Cardiovascular activity in an affectively neutral environment following inactivation of either aMCC or pMCC. No significant effect was detected in any of the cardiovascular measures analyzed. Data are displayed as mean ± SEM

Region	Infusion	HR	MAP	RMSSD	CSI	CVI	CVI/CSI
aMCC	Control	254.09 ± 22.03	96.78 ± 3.60	14.66 ± 2.08	2.85 ± 0.46	3.59 ± 0.12	1.54 ± 0.34
	Inactivation	258.61 ± 19.89	94.48 ± 4.30	16.23 ± 5.21	3.13 ± 0.65	3.53 ± 0.17	1.63 ± 0.50
pMCC	Control	253.13 ± 16.37	92.13 ± 5.74	14.10 ± 3.10	2.89 ± 0.36	3.54 ± 0.18	1.34 ± 0.22
	Inactivation	240.63 ± 12.60	88.71 ± 6.30	11.49 ± 2.29	3.51 ± 0.42	3.47 ± 0.17	1.05 ± 0.12

### Inactivation of aMCC Reduced the Expression of the Conditioned Threat Responses during the Extinction of a Pavlovian Conditioning Task

**Figure 5:**
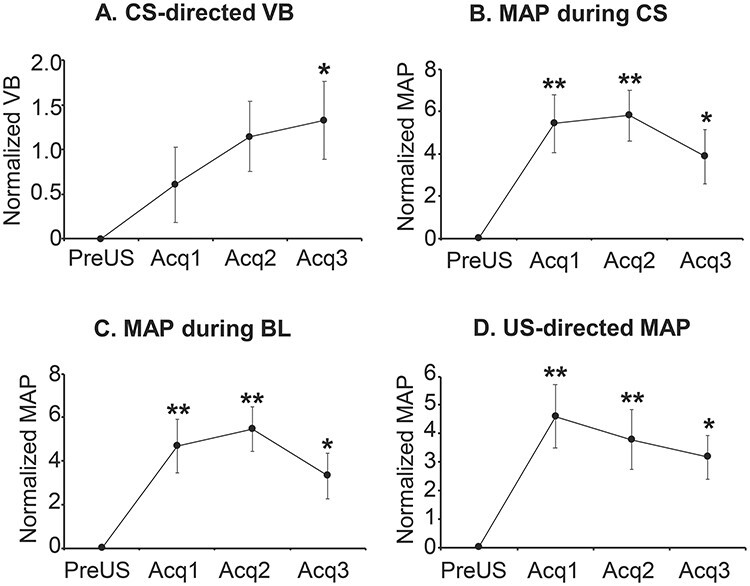
Mean responses across all acquisition sessions of Pavlovian threat conditioning. (*A*) CS-directed (CS-BL) vigilant behavior (VB), normalized to the PreUS period (before the threat, a rubber snake, is presented, US^−^). (*B*) Cardiovascular activity (MAP) during the CS period normalized to the PreUS period. (*C*) Cardiovascular activity (MAP) during the BL period normalized to the PreUS period. (*D*) US-directed (US–CS) cardiovascular activity (MAP) normalized to the PreUS period. Linear mixed-model ANOVA, post hoc contrasts comparing against PreUS ^*^P < 0.05, ^*^^*^P < 0.005.

**Figure 6:**
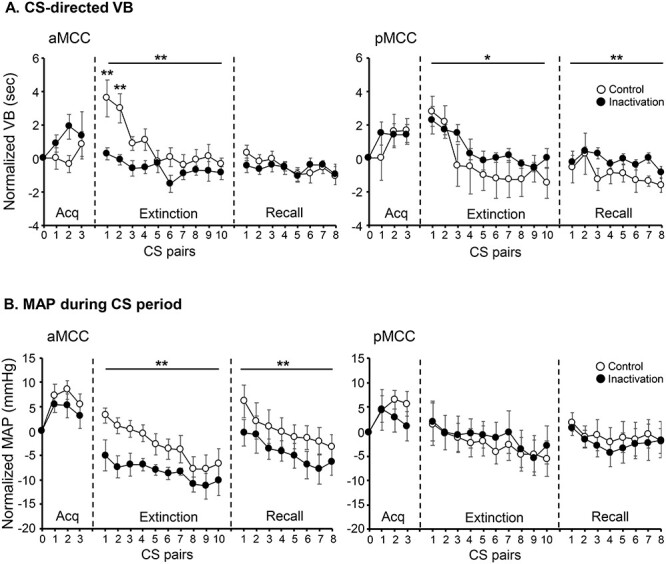
Mean responses across all sessions of Pavlovian threat conditioning for (*A*) CS-directed vigilant behavior (VB) and (*B*) cardiovascular activity (MAP) during the CS period, both normalized to the PreUS period (before the presentation of the snake, US^−^). Left panels show results for aMCC and right panels for pMCC. Empty circles correspond to control and filled circles to inactivation infusions. Linear mixed-model ANOVA, post hoc contrasts control versus inactivation, main effects are shown with a line above the curves, and specific CS pairs are indicated with asterisk above the point. ^*^P < 0.05, ^*^^*^P < 0.005.

During the acquisition phase of the Pavlovian threat conditioning task, when no manipulation took place, subjects showed a behavioral response directed specifically to the CS (CS-directed), which developed upon the presentation of the CS–US^+^ pairs (sound-snake) during the acquisition session (trials main effect *F*_3,101.04_ = 3.13, *P* = 0.029, Fig. 5*A*, mean across all acquisition sessions). The cardiovascular response after the presentation of the CS–US^+^ pairs was instead more generalized, evidenced by an increase of MAP during both the CS and BL periods (trials main effect *F*_3,99.62_ = 10.21, *P* = 6.35E-06 for CS period, and *F*_3,101.03_ = 10.70, *P* = 3.62E-06 for BL, Fig. 5*B* and *C*, respectively). A significant MAP response specifically to the snake (US^+^) over and above the CS response (US-directed) highlighted the animals’ arousal response to the US itself (trials main effect *F*_3,101.04_ = 8.17, *P* = 6.38E-05, Fig. 5*D*). During the extinction session, under normal conditions with only saline infused into the MCC, subjects showed a marked CS-directed VB and MAP response that were extinguished along the session (Fig. 6*A* and *B*, extinction phase). During the recall session, there was an overall decline in CS-directed VB and MAP responses across the session. While there was a clear recall of the conditioned MAP response at the start of the session, this was less so for the CS-directed VB response (Fig. 6*A* and *B*, recall phases). It can be noted that in both extinction and recall sessions, the CS-directed VB and MAP responses fall below the zero line as a consequence of the normalization to the PreUS period. For a detailed explanation, please see Supplementary Figure S1.

During the extinction phase, when MCC region-specific pharmacological inactivation took place, aMCC inactivation blunted both behavioral and cardiovascular responses to the proximal threat, while pMCC inactivation had the opposite effect primarily on the behavior (Fig. 6*A* and *B*, extinction phases). More specifically, for the CS-directed VB, a linear mixed-model ANOVA revealed a significant interaction between treatment and area (*F*_1, 264.70_ = 22.24; *P* = 3.90E-06; Fig. 6*A*). Compared with saline, inactivation of the aMCC reduced CS-directed VB (Fig. 6*A*, left, extinction phase, post hoc contrast *t*-ratio_265_ = −4.27; *P* = 0.0001), while inactivation of the pMCC showed a small although significant increase (Fig. 6*A*, right, extinction phase post hoc contrast *t*-ratio_265_ = 2.40; *P* = 0.017). For the cardiovascular response (MAP) during the CS period, there was a significant treatment by area interaction (*F*_1, 264.45_ = 17.55; *P* = 3.81E-05, Fig. 6*B*). Compared with control, inactivation of the aMCC reduced the MAP response during the CS period (Fig. 6*B*, left, extinction phase control vs. inactivation *t*-ratio _265_ = −4.78; *P* = 0.0001), while inactivation of the pMCC had no effect (Fig. 6*B*, right, extinction phase).

During the recall phase, which occurred the following day after the acute region-specific manipulation took place during the extinction, the analysis of CS-directed VB revealed a significant interaction between treatment and area (*F*_1, 208.90_ = 7.49; *P* = 0.007), with only animals that had received inactivation of pMCC the previous day, displaying an increased CS-directed VB response throughout the recall session (Fig. 6*A*, right, Recall phase post hoc contrast *t*-ratio_209_ = 3.40; *P* = 0.0008). For the cardiovascular response, the analysis showed a significant interaction between area and time (*F*_1, 200.25_ = 4.05; *P* = 0.046). Follow-up ANOVAs across CS pairs for each area indicated that this interaction was driven by a significant treatment effect for aMCC only (*F*_1, 90_ = 14.38; *P* = 0.0003), with animals that had received inactivation of aMCC the previous day, displaying a reduced MAP response during the CS period throughout the recall session (Fig. 6*B* left, recall phase post hoc contrast *t*-ratio_90_ = −3.79; *P* = 0.0003).

## Discussion

Taking into account potential anatomical and functional heterogeneity within the dACC with respect to its anterior (aMCC) and posterior (pMCC) extents and using specific paradigms to study the response to distal and proximal threat, this investigation revealed the differential contributions of these two subregions to the regulation of threat processing in the common marmoset. The aMCC was involved in both the behavioral response to distal threat associated with anxiety and the behavioral and cardiovascular responses in the regulation of conditioned, proximal threat associated with fear. In contrast, the pMCC played an opposite role but primarily in the behavioral responses to both uncertain, distal and certain, proximal threat.

Specifically, anxiety-like behaviors, displayed to an unfamiliar human acting as an uncertain distal threat, were reduced following inactivation of the aMCC. Detailed analysis of the behaviors that contributed to the overall anxiety score showed that this manifested primarily as a decrease in time spent at the top of the cage, away from the intruder, and a reduction in head and body bobs that are species-specific behaviors produced by marmosets at times of uncertainty. This was accompanied by an increase in locomotion, which is usually reduced in the presence of an unknown human. Conversely, inactivation of the pMCC, if anything, promoted an anxiogenic-like response as revealed by a trend toward an increase in overall anxiety-like scores, driven by significant reductions in time spent at the front and locomotion. These findings provide the first direct causal evidence for differential functional roles of these two subregions of the MCC in the regulation and expression of anxiety-like behaviors and the processing of threat. Of the various measures, only locomotion was commonly affected, showing opposite effects with respect to each subregion inactivation. Locomotion was also significantly affected following aMCC inactivation prior to the introduction of the intruder during the separated phase, which could also be perceived as a mildly threatening context given that the animals are usually divided from their cage mates only when they need to receive a certain procedure (e.g., weighing, transport to behavioral apparatus, and injections). Of particular significance were the behaviors that were differentially altered by inactivation of aMCC and pMCC, which may provide insight into the different defensive strategies engaged by these two subregions in response to threat. While aMCC appears involved in behaviors of avoidance (inducing upward flight) and vigilance (increasing bobs) associated with the anxiety-like response (Quah et al. 2020), pMCC may play a role in threat appraisal and risk assessment by approaching the threat (increasing time spent at the front, close to the intruder) (Blanchard et al. 2011). This is consistent with previous findings in macaques, using a similar HI test, in which activity within the more caudal regions of the dACC, likely mapping onto pMCC, correlated with the ability to regulate the freezing response in relation to the presence of the threat stimulus (Kalin et al. 2005).

In addition to the opposing contributions of both aMCC and pMCC to the behavioral response to uncertain, distal threat, these subregions also showed opposing roles in the regulation of the reactivity to proximal threat in the Pavlovian threat conditioning and extinction paradigm. Specifically, aMCC inactivation immediately prior to the extinction session blunted the recall and expression of the conditioned behavioral response, while pMCC inactivation had an opposite effect increasing the reactivity to the conditioned stimuli. The behavioral blunting observed after aMCC inactivation was accompanied by a similar blunting of cardiovascular response during the CS period, the latter carrying over into the Recall session the next day. This was not the case for the pMCC inactivation that showed no effect on cardiovascular reactivity to proximal threat during the CS period. Thus, these results provide causal evidence in support of the correlative functional imaging findings that implicate activity in the aMCC with the reactivity to proximal threat in humans (Milad et al. 2007; Mobbs et al. 2010; Qi et al. 2018). Together with the reduction in distal threat reactivity, these findings reveal the major role of the aMCC in facilitating an animal’s reactivity to threat.

While our findings are also consistent with the proposal that the dACC, including the MCC, integrates cardiovascular regulation with volitional behavior (Critchley 2005; Critchley and Harrison 2013), they rule out a role for either the aMCC or pMCC in the regulation of basal cardiovascular activity per se. No significant effects were observed upon inactivation of either subregion on measures of HR, MAP, or HRV. This contrasts with the marked impact that both inactivation and activation of a subgenual region of the anterior cingulate, area 25, have on basal cardiovascular activity, which we showed increases and reduces HRV by tipping the balance toward parasympathetic and sympathetic control, respectively (Wallis et al. 2017; Alexander et al. 2020).

Until now, the causal roles of subregions of the primate dACC along the rostrocaudal axis have not directly been examined. Given that this region is consistently implicated in emotion regulation (Etkin et al. 2011) and abnormalities are observed in anxiety disorders (Etkin and Wager 2007; Shin et al. 2009), the present findings of a functional dissociation between the aMCC and pMCC with regards to negative emotion regulation could have major implications for our understanding of these disorders. Here, we causally implicate the aMCC in both the behavioral expression of distal threat response (associated with anxiety) as well as the cardiovascular and behavioral expression of proximal conditioned threat response (associated with fear), suggesting a broad role in the regulation of negative emotion. Such a role is consistent with the connectivity of the aMCC (more so than the pMCC) with the basolateral amygdala, dorsal hypothalamus, and dorsolateral periaqueductal gray (Carmichael and Price 1995; An et al. 1998; Öngür et al. 1998), which are involved in coordinating visceral and behavioral responses to escapable stressful situations with proactive or reactive response strategies, for example, threat display, fight or flight, hypertension, and tachycardia (Bandler et al. 2000; Mobbs et al. 2020). Moreover, anterior cingulotomy, in which the aMCC is ablated, has been reported to be effective in reducing symptoms of anxiety disorders (Ballantine et al. 1987). The present findings, selectively targeting the aMCC, provide direct support for the hypothesis that normalizing activity in the aMCC, perhaps using deep brain stimulation (Neumaier et al. 2017), might be an effective strategy for treating anxiety.

In contrast, the role of the pMCC in regulating negative emotion is less clear. While its inactivation seems to increase anxiety-like behaviors in response to distal, uncertain threat, it had an effect primarily on the behavioral expression of proximal, conditioned threat response. While activity in aMCC has been implicated in the cognitive processing of emotion-related information, pMCC activity seems not to be related to affect (Vogt et al. 2003; Vogt 2005). It has been proposed that a primary role of pMCC is reflexive orientation of the body in space to sensory stimuli, including noxious ones (Vogt 2005). In contrast to the aMCC, the pMCC receives parietal afferents (Vogt and Pandya 1987), specifically from a subregion of the inferior parietal cortical convexity, area PG, corresponding to Brodmann area 7 and Vogt area 7a (Gregoriou et al. 2006). This region, in turn, is the target of visual somatosensory and auditory inputs (Rozzi et al. 2006) contributing to the construction of representations of surrounding space, for space perception and guidance of motor behavior. It has been hypothesized that this multisensory information is provided to the pMCC in order to control body orientation and reflexive movements toward the stimulus, as discussed in Vogt (2016). Moreover, this parietal region itself is part of the executive control network (also known as frontoparietal network) that is critical for coordinating behavior in a rapid and accurate goal-driven manner (Marek and Dosenbach 2018; Shen et al. 2020). Thus, it is possible that the pMCC may be involved in cognitive processes associated with the spatiotemporal characteristics of the threat and the control of the subsequent behavioral response. As such, even within the constraints of the behavioral testing apparatus during the Pavlovian threat conditioning paradigm used in the present study, we were able to detect behavioral changes after pMCC inactivation, as well as detecting them in the HI test in which a broad repertoire of behaviors can be enacted within the home cage environment. The lack of effect on the regulation of cardiovascular response to the conditioned threat stimuli most likely reflects the fact that the pMCC is not activated by emotionally related stimuli per se and does not have the appropriate connections to cardiovascular effector centers (Vogt 2005).

Processing of negative emotion is just one of several functions that have been attributed to the dACC, including cognitive control, action selection, and pain (Lieberman and Eisenberger 2015; Kolling et al. 2016; Shenhav et al. 2016). It is likely that these distinct functions involve related interacting processes and that functional distinctions between aMCC and pMCC exist beyond the domain of negative emotion. However, here, we provide causal evidence for the distinct functional roles of the aMCC and pMCC within the negative emotion domain, specifically with respect to reactivity to threat. Like subcallosal cingulate area 25 (Alexander et al. 2020), aMCC is implicated in how an animal reacts to both proximal and distal threat with activity in both regions promoting sensitivity to threatening stimuli, while only subcallosal area 25 contributes to the regulation of basal levels of cardiovascular activity, per se. This should be contrasted with findings that prefrontal, as opposed to cingulate, regions are specifically implicated in distal, uncertain, rather than proximal, certain threat purportedly when there is time to engage a range of attentional and cognitive strategies to inform decision-making (Mobbs et al. 2020), as highlighted in human neuroimaging studies (Qi et al. 2018) and more recently substantiated in neuropsychological studies in marmosets (Roberts 2020; Stawicka et al. 2020). Altogether, these findings have widespread implications for our understanding of the mixed etiology of anxiety disorders, in which the regulation of negative emotion is impaired, as well as implications for our understanding of the structural and functional organization of the dACC.

## Funding

Medical Research Council Programme (grant MR/M023990/1 to A.C.R.). S.R. was funded by a Biotechnology and Biological Sciences Research Council Doctoral Training Programme studentship, and N.H. was supported by a Wellcome Trust Senior Investigator Award (grant 104631/Z/14/Z to T.W.R.).

## Notes

We thank the University of Cambridge Biological Services staff for their care of the marmosets throughout the study, and Benjamin Phillips for advice on statistical analyses. *Conflict of Interest*: None declared.

## Supplementary Material

Supplementary_material_FINAL_bhab121

## List of abbreviations

ACd, dorsal anterior cingulate; aMCC, anterior MCC; BL, baseline, 20 seconds before the CS; CS, conditioned stimulus; CSI, cardiac sympathetic index; CVI, cardiac vagal index; dACC, dorsal anterior cingulate cortex; dBP, diastolic blood pressure; EFA, exploratory factor analysis; HI, human intruder test; HR, heart rate; HRV, heart rate variability; IBIs, interbeat intervals; MAP, mean arterial pressure; MCC, midcingulate cortex; NFP SMI-32, neurofilament protein SMI-32; NHP, nonhuman primates; pACC, pregenual anterior cingulate cortex; pMCC, posterior MCC; PreUS, trials previous to the presentation of the US; rCG1/rCG2, rostral anterior cingulate cortex; RMSSD, square root of the mean squared standard deviation; sBP, systolic blood pressure; TSAB, time spent at the back; TSAF, time spent at the front; US, unconditioned stimulus; VB, vigilant behavior; vITI, variable intertrial interval
