# Supplementary material for: Differential Contribution of Anterior and Posterior Midcingulate Subregions to Distal and Proximal Threat Reactivity in Marmosets
Source: Cereb Cortex. 2021 Jun 1;31(10):4765–80. doi: 10.1093/cercor/bhab121 (PMC8408452; doi:10.1093/cercor/bhab121)
Supplement: Supplementary_material_FINAL_bhab121 [file supplementary_material_final_bhab121.docx]

**Supplementary material for:**

Title: **Differential contribution of anterior and posterior midcingulate subregions to distal and proximal threat reactivity in marmosets**

Sufia S Rahman^1,3^, Kevin Mulvihill^1,^ Christian M Wood^1,3^, Shaun KL Quah^1,3^, Nicole K Horst^2,3^, Hannah F Clarke^1,3^, Gemma J. Cockcroft^1,3^, Andrea M Santangelo^1,3*†^ and Angela C Roberts^1,3*^

^1^ Department of Physiology, Development and Neuroscience, University of Cambridge

^2^ Department of Psychology, University of Cambridge

^3^ Behavioral and Clinical Neuroscience Institute, University of Cambridge

^*^ Joint senior author

^†^Corresponding author: Andrea M. Santangelo, Department of Physiology, Development and Neuroscience, University of Cambridge, UK.

Telephone: +44 1223 339 012

Email: as966@cam.ac.uk

**Content:**

**Supplementary tables I-IV**

**Supplementary figure 1**

| **Suppl. Table I:** Human intruder test, order of infusion type (Sal/Mb) per area (aMCC/pMCC) | | | | | | | | | | | | |
| --- | --- | --- | --- | --- | --- | --- | --- | --- | --- | --- | --- | --- |
| Subject | Ja | To | G* | A | S | Wa | Tr* | Ba | Bu | Ju | W-e | Ye |
| Test 1 | Sal aMCC | Sal pMCC | Sal pMCC | Sal pMCC | Sal pMCC | Sal aMCC | Sal aMCC | Sal aMCC | Sal aMCC | Sal pMCC | Sal aMCC | Sal pMCC |
| Test 2 | Mb aMCC | Mb pMCC | Mb pMCC | Mb pMCC | Mb pMCC | Mb aMCC | Mb aMCC | Mb aMCC | Mb aMCC | Mb pMCC | Mb aMCC | Mb pMCC |
| Test 3 | Sal pMCC | Sal aMCC | Sal pMCC | Sal aMCC | Sal aMCC | Sal pMCC | Sal aMCC | Sal pMCC | Sal pMCC | Sal aMCC | Sal pMCC | Sal aMCC |
| Test 4 | Mb pMCC | Mb aMCC |  | Mb aMCC | Mb aMCC | Mb pMCC |  | Mb pMCC | Mb pMCC | Mb aMCC | Mb pMCC | Mb aMCC |
| Test 5 | Sal pMCC | Sal aMCC |  | Sal pMCC | Sal aMCC | Sal pMCC |  | Sal pMCC | Sal aMCC | Sal aMCC | Sal pMCC | Sal aMCC |
| Sal: saline (control), Mb: muscimol/baclofen (inactivation), *second cannula placement outside MCC | | | | | | | | | | | | |

| **Suppl. Table II:** Pavlovian Threat Conditioning, order of infusion type (Sal/Mb) per area (aMCC/pMCC) | | | | | | | | |
| --- | --- | --- | --- | --- | --- | --- | --- | --- |
| Subject | Ja | To | G* | A | S | Wa | Tr* | Ba |
| Block 1 | Sal aMCC | Sal pMCC | Sal pMCC | Sal pMCC | Sal aMCC | Sal aMCC | Sal aMCC | Sal aMCC |
| Block 2 | Mb aMCC | Mb pMCC | Mb pMCC | Mb pMCC | Mb aMCC | Mb aMCC | Mb aMCC | Mb aMCC |
| Block 3 | Sal pMCC | Sal aMCC |  | Sal aMCC | Sal pMCC | Sal pMCC |  | Sal pMCC |
| Block 4 | Mb pMCC | Mb aMCC |  | Mb aMCC | Mb pMCC | Mb pMCC |  | Mb pMCC |
| Sal: saline (control), Mb: muscimol/baclofen (inactivation), *second cannula placement outside MCC | | | | | | | | |

| **Suppl. Table III: Human Intruder Test, Intruder phase** | | | |
| --- | --- | --- | --- |
| **Region** | **Measures** | **Control** | **Inactivation** |
| **aMCC** | **EFA^a^** | -0.44±0.29 | -1.20±0.22 |
|  | **Locomotion (sec)^b^** | 6.32±1.02 | 9.88±1.06 |
|  | **TSAF (%)** | 53±7 | 59±4 |
|  | **TSAB (%)** | 14±3 | 15±3 |
|  | **Height (cm) ^b^** | 44.95±3.40 | 37.39±2.72 |
|  | **Bobs ^b^** | 8.25±3.06 | 3±2.22 |
|  | **Tse-Egg calls** | 4.20±1.22 | 2.50±1.24 |
|  | Tsik calls | 1.95±1.23 | 1±0.49 |
|  | Tsik-Egg calls | 7.95±3.83 | 0.70±0.47 |
|  | Egg calls | 2.55±0.84 | 1.60±0.90 |
| **pMCC** | **EFA** | -0.16±0.31 | 0.30±0.34 |
|  | **Locomotion (sec)^b^** | 6.09±0.90 | 4.36±0.63 |
|  | **TSAF (%)^b^** | 47±7 | 33±7 |
|  | **TSAB (%)** | 14±3 | 19±5 |
|  | **Height (cm)** | 48.74±3.29 | 49.56±3.78 |
|  | **Bobs** | 10.28±3.42 | 12.44±5.07 |
|  | **Tse-Egg calls** | 2.28±1.06 | 1.89±0.72 |
|  | Tsik calls | 0.94±0.69 | 1.67±1.28 |
|  | Tsik-Egg calls | 6.67±3.62 | 9.89±5.93 |
|  | Egg calls | 4.67±1.60 | 5.67±1.86 |
| ^a^ ANOVA Treatment x Area F_1, 22.97_ = 14.62; p=.0009, aMCC saline vs inactivation pairwise comparison p<.005, ^b^ two-tailed, paired t-test saline vs inactivation p<.05 | | | |

| **Suppl. Table IV: Human Intruder Test, Separated phase** | | | | |
| --- | --- | --- | --- | --- |
| **Region** | **Measure** | **Control** | **Inactivation** | |
| **aMCC** | Locomotion (sec)^a^ | 9.38±1.81 | 13.98±2.55 | |
|  | TSAF (%) | 48.33±7.16 | 45.93±6.18 | |
|  | TSAB (%) | 19.19±5.29 | 25.31±5.76 | |
|  | Height (cm) | 46.51±2.68 | 46.37±3.45 | |
| **pMCC** | Locomotion (sec) | 9.52±2.29 | 8.18±1.22 | |
|  | TSAF (%) | 39.23±6.18 | 39.89±6.82 | |
|  | TSAB (%) | 22.73±5.04 | 33.72±9.28 | |
|  | Height (cm) | 49.86±3.28 | 51.85±4.39 | |
| ^a^ two-tailed, paired t-test saline vs inactivation p<.05 | | | |  |

**Supplementary Figure 1: Pavlovian conditioning threat task**

**Supplementary figure 1:** Raw data for cardiovascular responses (MAP) during the Pavlovian conditioning threat task. Note that animals only receive MB on the extinction session but are still labelled MB during Acquisition before, and Extinction Recall after, the extinction session. The MAP levels of all animals show reductions during extinction (see Ext10) and extinction recall (see Rec8) that go below baseline as indexed by the pre-US MAP level during acquisition (shown with a dashed line). The reason for animals seemingly going below baseline is dependent on the baseline we have chosen to use for this analysis. This baseline is their MAP levels in the prior acquisition session before receiving exposure to the snake and associated conditioned stimulus. However, the animals during this period are exposed to a novel stimulus and opening of the door into the chamber, all of which are necessary as controls for the Pavlovian conditioning that follows, but which act to induce a mild level of unconditioned arousal in their own right. This is apparent when comparing the pre-US raw levels with the levels in the neutral conditions (**Table 2,** and also added to this figure for comparison purposes) which clearly show that PreUS levels are higher than levels in the neutral condition. Importantly, though, as can also be seen in **Fig 6**, MAP levels rise even more when the animal experiences the specific CS-US pairing (Acq3). During extinction, levels are above baseline initially due to recall of the conditioned response (Ext1). However, these then decline and by the end of the extinction (Ext10) and recall extinction sessions (Rec1 and Rec8) approach levels seen in the neutral condition. Based on this, the only reason the aMCC inactivation group appear below baseline even at the start of extinction is because the reduction in threat reactivity induced by aMCC inactivation causes their MAP levels to approach the levels they display in the neutral condition when they are relaxed, starting lower than their levels during the pre-US of acquisition when they are not under the drug and show some arousal because of the novel stimulus and opening doors.
